# Supplementary material for: FastqPuri: high-performance preprocessing of RNA-seq data
Source: BMC Bioinformatics. 2019 May 3;20:226. doi: 10.1186/s12859-019-2799-0 (PMC6500068; doi:10.1186/s12859-019-2799-0)
Supplement: Supplementary file 2 — Archive of FastqPuri. Archive containing all files needed to install and run FastqPuri v1.0.6. Date stamp March 22, 2019. (GZ 47,819 kb) [file 12859_2019_2799_MOESM2_ESM.gz › FastqPuri-1.0.6/html/globals_i.html]

FastqPuri: File Members


FastqPuri


Here is a list of all documented file members with links to the documentation:

### - i -

- ignore\_line()
  : fa\_read.c
- Info
  : stats\_info.h
- init\_alLUTs()
  : adapters.h
  , adapters.c
- init\_Bfilter()
  : bloom.c
  , bloom.h
- init\_Bfkmer()
  : bloom.h
  , bloom.c
- init\_DSadap()
  : trimDS.c
  , trimDS.h
- init\_entries()
  : fa\_read.c
- init\_fa()
  : fa\_read.c
- init\_info()
  : stats\_info.c
  , stats\_info.h
- init\_LUTs()
  : bloom.h
  , bloom.c
- init\_map()
  : Lmer.c
  , Lmer.h
- insert\_and\_fetch()
  : bloom.c
  , bloom.h
- insert\_entry()
  : tree.c
  , tree.h
- insert\_Lmer()
  : tree.c
  , tree.h
- Iparam\_makeBloom
  : init\_makeBloom.h
- Iparam\_makeTree
  : init\_makeTree.h
- Iparam\_Qreport
  : init\_Qreport.h
- Iparam\_Sreport
  : init\_Sreport.h
- Iparam\_trimFilter
  : struct\_trimFilter.h
- is\_read\_inBloom()
  : trim.c
  , trim.h
- is\_read\_inTree()
  : trim.h
  , trim.c


---

Generated on Mon Mar 19 2018 23:42:01 for FastqPuri by  

 1.8.14
